# Prospective Randomized Phase II Parallel Study of Vinorelbine Maintenance Therapy versus Best Supportive Care in Advanced Non-Small Cell Lung Cancer

**Published:** 2017

**Authors:** Adnan Khosravi, Zahra Esfahani-Monfared, Sharareh Seifi, Kian Khodadad

**Affiliations:** 1 Tobacco Prevention and Control Research Center, National Research Institute of Tuberculosis and Lung Diseases (NRITLD), Shahid Beheshti University of Medical Sciences, Tehran, Iran; 2 Chronic Respiratory Diseases Research Center, National Research Institute of Tuberculosis and Lung Diseases (NRITLD), Shahid Beheshti University of Medical Sciences, Tehran, Iran; 3 Dalhousie University, Cape Breton Cancer Centre, Sydney, Nova Scotia, Canada.

**Keywords:** Lung cancer, Maintenance chemotherapy, Vinorelbine

## Abstract

**Background:**

Maintenance strategy has been used to improve survival in non-small cell lung cancer (NSCLC). We investigated whether switch maintenance therapy with vinorelbine improved progression free survival (PFS) after first-line chemotherapy with gemcitabine plus carboplatin.

**Materials and Methods:**

In this single blind, parallel, phase 2, randomized trial, patients with NSCLC pathology, age >18 years, Eastern Cooperative Oncology Group (ECOG) performance status (PS) score of 0–2, and advanced stage (IIIB and IV) were treated with up to 6 cycles of gemcitabine 1250 mg/m^2^ (day 1 and 8) plus carboplatin AUC 5 (day 1) every 3 weeks. Patients who did not show progression after first-line chemotherapy were randomly assigned to receive switch maintenance with vinorelbine (25 mg/m^2^, day 1, 15) or the best supportive care until disease progression.

**Results:**

A total of 100 patients were registered, of whom 34 had a non-progressive response to first-line chemotherapy and randomly received maintenance vinorelbine (n=19) or best supportive care (n=15). The hazard ratio of PFS in the vinorelbine group relative to the best supportive care group was 1.097 (95% confidence interval = 0.479–2.510; P-value =0.827). There was no significant difference between the overall survival for the two groups (P=0.068).

**Conclusion:**

Switch maintenance strategies are beneficial, but defining the right candidates for treatment is a problem. Moreover, the trial designs do not always reflect the real-world considerations. Switch maintenance therapy with vinorelbine, though had tolerable toxicity, did not improve PFS in patients with NSCLC. Therefore, other agents should be considered in this setting.

## INTRODUCTION

Since 1987, lung cancer has been the leading cause of cancer-related deaths in women and men worldwide. (1) In Iran (situated in southwest Asia / Middle East), lung cancer ranks 2^nd^ in men and 3^rd^ in women as the cause of cancer-related death (2). Nearly, 85% of newly diagnosed lung cancers have a non-small cell lung cancer (NSCLC) pathology (3, 4), and are locally advanced (inoperable stage IIIB) or metastatic (stage IV) at the time of diagnosis (5). Systemic standard platinum-based chemotherapy as first-line treatment is recommended for patients with advanced stage disease (6). With combination platinum-based chemotherapy regimens, the median of overall survival (OS) and median progression free survival (PFS) are 8–11 and 4 months, respectively (7). Recently, maintenance strategies, which are defined as opportunities for extending the duration of first-line treatment (continuing one or all the drugs previously administered as first-line) or switching to a different and non-cross-resistant agent, are introduced immediately after completion of first-line treatment. They have received great attention, especially in patients who benefit from the initial treatment, in order to prolong the duration of disease control (8,9). Administration of a single agent as maintenance therapy following 4–6 cycles of combination chemotherapy has been studied in some randomized clinical trials. Gemcitabine, docetaxel, vinorelbine, paclitaxel, and gefitinib have been administrated in maintenance settings, and some of them have shown improvement in OS (10–14). In contrast, a meta-analysis of randomized trials demonstrated significantly increased PFS but not OS by using the maintenance strategy (15). Navelbine (vinorelbine, NVB) is the first semi synthetic 5′-nor-vinca-alkaloid that is manufactured from alkaloids extracted from the rosy periwinkle, *Catharanthus roseus* (16). It has been shown to have a good level of activity in different solid tumors (17). Vinorelbine Tartrate alone and in combination with other cytotoxic or targeted agents are approved by the Food and Drug Administration (FDA) for the treatment of NSCLC (18, 19). It has also been used as second-line chemotherapy in progressive disease (20).

We designed this study to examine whether vinorelbine switch maintenance therapy would improve PFS in patients with advanced stage NSCLC whose disease had not progressed after the initial treatment with gemcitabine plus carboplatin.

## MATERIALS AND METHODS

This survey is a parallel, randomized, prospective, phase 2, single blind study with 100 NSCLC histologically confirmed patients at advanced stage (IIIB or IV) (21). These patients were referred to the National Institute of Tuberculosis and Lung Disease (NRITLD), Masih Daneshvari Hospital, a referral hospital in Tehran, the capital of Iran. The stratified random sampling method was used, and the allocation ratio was 1: 1. A physician performed generation of random allocation sequence, enrolling participants and assigning participants to interventions. In this study, only the investigator was aware of the group assignment (single blind). Informed written consent was obtained from all participating patients prior to the study according to Shahid Beheshti Medical University’s ethics and scientific committee’s guidelines in compliance with the Helsinki Declaration. This trial has been registered in the Iranian Clinical Trial Registration (ICTR) (Trial number: IRCT2015060822610N1).

### Eligibility criteria:

Inclusion criteria were: NSCLC pathology, age of 18 years or older, Eastern Cooperative Oncology Group (ECOG) performance status (PS) score of 0–2, (22) no previous history of any systemic chemotherapy and advanced stage disease according to the American Joint Committee for Cancer Staging (AJCC), 7th edition (21). Other eligibility criteria included at least one unidimensionally measurable or assessable disease, adequate bone marrow reserve, serum creatinine less than or equal to 1.5 mg/dL or a calculated creatinine clearance greater than or equal to 60 mL/min, bilirubin level less than or equal to 2.0 mg/dL, AST less than or equal to twice the institutional upper limits of normal, or less than or equal to four times the institutional upper limits of normal if the patient had liver metastasis. Exclusion criteria included: administration of systemic chemotherapy, PS 3 and 4 and tumor histology that was small cell lung cancer (SCLC) or metastatic from other sites. Patients with significant or uncontrolled cardiac, metabolic, or infectious diseases or with symptomatic brain metastasis were excluded.

### Trial design:

This study had two phases: The primary phase included first-line chemotherapy with intravenous gemcitabine 1250 mg/m^2^ (day 1 and 8) plus carboplatin AUC 5 (day 1) every 3 weeks. In the absence of progressive disease or intolerable toxicity, the patients were treated for a minimum of four cycles. Patients were evaluated after each cycle for any response based on a physical exam and chest X-ray. Chest computed tomography (CT) scan was requested after every 2 cycles and/or at the termination of the protocol. Patients who achieved a complete (CR) or partial response (PR) received two additional cycles of therapy, for a maximum of 6 cycles. Response evaluation was assessed according to the RECIST 1.0 guideline (23). The secondary phase included a randomized maintenance phase with vinorelbine (Navelbine, Pierre Fabre) 25 mg/m^2^ (day 1, 15) given up to disease progression or the best supportive care. Patients were eligible for the maintenance phase if they had received at least four cycles of first-line chemotherapy with gemcitabine plus carboplatin with documented evidence (clinically and radiologically) of non-progressive tumor response (CR, PR or stable disease). Best supportive care was defined as palliative non-cancer therapy given at the investigator’s discretion.

Base line tumor measurements were performed less than 2 weeks before the first course of maintenance therapy with CT scan or MRI. All patients were evaluated by physical examination, which included a complete blood cell (CBC) count and biochemistry study, prior to each therapy. Dose modification and concomitant G-CSF were allowed during the treatment course based on the grade of neutropenia. The dose of the cytotoxic agent was attenuated by 25% if patients experienced neutropenia (1,000–1,500/dL) and/or had a platelet count of 75,000–100,000/dL. If the neutrophil or platelet count was less than 1,000/dL and 75,000/dL, respectively chemotherapy was postponed. Notably, in this real world study, the dosage of the cytotoxic agent was adjusted by the clinicians based on the patient’s age, frailty, or other adverse events during the course of treatment. However, any patient who developed a severe reaction was taken off the protocol.

Toxicity assessment was based on the “Common Terminology Criteria for Adverse Events” (CTCAE) version 3.0 (24). Criteria for withdrawal from the study included unacceptable toxicity as determined by the treating physician in consultation with the study coordinator, a delay in treatment greater than 2 weeks, requirement for palliative radiotherapy, or patient refusal.

### Statistical analysis :

The primary end point of this phase 2 study was PFS. The secondary objectives were OS and adverse events.

All confidence intervals (CIs) for parameters to be estimated were constructed with a significance level of alpha = 0.05 (a 95% confidence level). Patients were assigned to the vinorelbine (n=19) or best supportive care (n=15) group, after being centrally randomized to a 1:1 ratio during the 3rd week after the first- line chemotherapy using the Stata 9.0 (StataCorp, College Station, TX, USA) statistical software.

For testing the differences in the categorical variables between the two groups, the chi-square test or Fisher’s exact test was used. The difference in the quantitative variables of the two groups was compared using the Student’s t-test or non-parametric Mann-Whitney test. We tested the hypothesis that an 18 months survival rate could be expected in 10 % of the best supportive care group and 20% of the maintenance group, using a sample size that was determined using a significance level of 5% for alpha. Kaplan Meier’s survival curves were obtained, and the log-rank test was used to assess the significance of differences in PFS and OS between the two study groups. PFS was calculated from the date of registration in maintenance phase to the date of progression or death. OS was calculated from the date of registration in maintenance phase to the date of death. A COX-PH regression model was used to estimate the hazard ratios and their 95% CIs (confidence intervals).

34 patients were finally randomized after assuming an accrual period of 3 years; a potential follow- up for 2 years for the last patients and a type I error rate of 0/5. The study was stopped on March 20, 2013. All analysis was performed using SPSS version 21.

## RESULTS

### Patient characteristics and Initial treatment:

**Figure 1.**
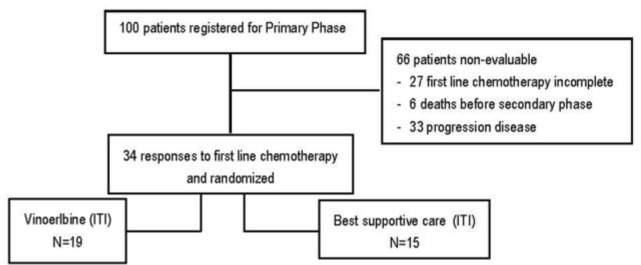
Diagram of the study; clinical trial flow chart. A total of 100 patients received study treatment consisting of at least 4 cycles firstline chemotherapy with Gemcitabine and carboplatin with non-progressive response and randomized for phase 2.; ITI: intent to treatment.

A total of 100 patients were enrolled in this study (Figure 1). The mean age of the patients was 59.73 years. Table 1 shows the other patient characteristics at baseline in the primary and secondary phases. Most of the patients were men, smokers and with advanced stage disease (Table 1). No significant difference was noted in any of the characteristics listed between the two groups including age, sex, smoking status, stage and histologic subtype. Of the 100 patients for whom first-line chemotherapy treatment data were available, 6 died before the evaluation, 27 patients refused to continue treatment and 33 of them showed disease progression. Thirty-four patients responded to induction chemotherapy, with 15 of the 34 (44.1%) cases demonstrating PR and 19 (55.9%) showing stable disease (SD). No CR was reported. All of the 34 patients, who responded to initial chemotherapy, were randomly assigned to the vinorelbine (19 patients) or best supportive care (15 patients) groups.

**Table 1.** Demographic characteristics of patients in secondary phase of trial.

**Patient characteristics**	**Registered**	**Maintenance Vinorelbine group, n (%)**	**Best supportive care group, n (%)**	**P-value**
**Mean age, year(range)**	59.73(33–82)	60.53(48–75)	64.73(40–82)	0.180
**Gender**				
*Female*	25(25%)	6(31.6%)	4(26.4%)	>0.999
*Male*	75(75%)	13(68.4%)	11(73.3%)	
**Histology**				
*Adenocarcinoma*	51(51%)	10(52.6%)	7(46.6%)	0.170
*SCC†*	27(27%)	3(15.8%)	6(40%)	
*Undetermined NSCLC‡*	20(20%)	6(31.5%)	2(13.3%)	
*Large cell Carcinoma*	2(2%)	-	-	
**Smoking status**				
*Yes*	66(66%)	8(42.1%)	10(66.7%)	0.154
*No*	34(34%)	11(57.9%)	5(33.3%)	
**Stage**				
*IIIB*	11(11%)	6(31.6%)	2(13.3)	0.257
*IV*	89(89%)	13(68.4%)	13(86.7%)		

† SCC: Squamous cell carcinoma,

‡ NSCLC: Non-small cell lung cancer

Six deaths (6%) occurred after the first-line treatment that was unrelated to the induction chemotherapy (five were due to severe physical state alteration and one was an unknown cause).

### Maintenance treatment delivery

The number of vinorelbine cycles administered was as follows: one to three in 10 patients, four to eight in 7 patients and nine in 2 patients. The mean duration of vinorelbine chemotherapy was 10 weeks, and the median of total delivered dose was 240 mg (range 100–1090 mg). Vinorelbine was stopped due to progressive disease in 14 patients (73.6%), toxicity in 1 patient (5.2%), treatment refusal in 3 patients (15.7%) and death from an unknown cause in 1 patient (5.2%).

### Toxicity

The grade 3 toxicities that occurred in at least 33.3% of patients are listed in Table 2. One patient in the maintenance group was not assessable for toxicity. Grade 4 toxicity was not noted in any of the patients. The most frequent toxicity was hematologic. No patients from either group developed febrile neutropenia, nausea, and vomiting, sepsis, pulmonary toxicity, and thrombocytopenia. Grade 3 leukopenia, anemia, and peripheral neuropathy were observed in 16.6%, 33.3%, and 16.6% of patients, respectively. In one patient maintenance therapy was terminated due to grade 3 peripheral neuropathy and in two patients, the dose of vinorelbine was adjusted for leukopenia. No death was reported due to toxicity.

**Table 2.** Drug-related toxic effects

	**Maintenance group (n=18)**		**Best supportive care group (n=15)**	
	Grades 2 or 3	All grades	Grades 2 or 3	All grades
**Haematologic toxicities**				
Anemia	0	0	6(33.3%)	8(44.4%)
Leukopenia	0	0	3(16.6%)	5(27.7%)
**Non-haematologic toxicities**				
Sensory neuropathies	0	0	3(16.6%)	3(16.6%)

### Response in maintenance group

Of the 19 patients in the vinorelbine group, PR was seen in 4 (21.05%) patients. No patients achieved CR, 12 (63.1%) patients had SD, and in two patients (10.5%) progression was seen after the first course of chemotherapy.

**Figure 2.**
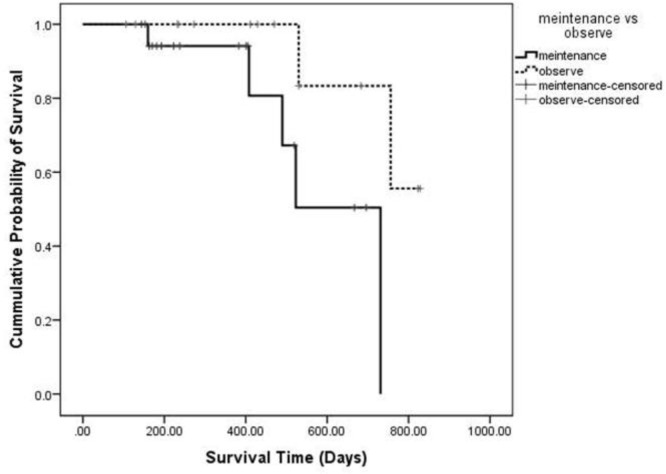
Kaplan-Meier survival curve for Overall survival (OS) in Maintenance therapy vs Best supportive care. Non-significant differences was observed between Maintenance therapy group and Best supportive care group. (P=0.068).

The mean follow-up times from the date of randomization were 4.45 and 6.29 months in the vinorelbine and best supportive care groups, respectively. The hazard ratio of OS in the vinorelbine relative to the best supportive care was 7.62 (95% CI = 0.862–67.399; P-value =0.068) (Figure 2).

**Figure 3.**
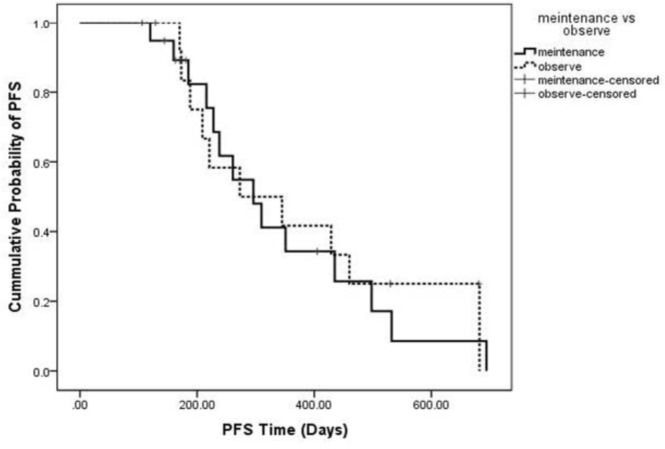
Kaplan-Meier survival curve from onset of recurrence for the effect of Maintenance therapy on progression free survival. Non-significant shortened PFS was observed in Best supportive care group. (P=0.897)

The hazard ratio of PFS was 1.097 (95% CI = 0.479–2.510; P-value =0.827) (Figure 3). The median PFS was 9.8 months in the maintenance group and 9.1 in the other group.

Among patients who had PR response to first-line chemotherapy, median PFS was 8.7 and 14.3 months in the vinorelbine and best supportive care groups, respectively (P=0.739). The median PFS in patients with SD in response to initial chemotherapy was 9.8 and 6.2 in the vinorelbine and best supportive care groups, respectively (P=0.854).

Post-maintenance or best supportive care therapies used are listed in Table 3. Three patients in the best supportive care group and two patients in the vinorelbine group were not assessable due to discontinuation of Vinorelbine therapy.

**Table 3.** Summary of post- discontinuation therapy

**Post–discontinuation therapy**	**Maintenance group(n=19)**	**Best supportive care group(n=15)**	P*-value
**RT^a^**	2(10.5%)	1(6.6%)	0.634
**Chemotherapy**			
Docetaxel	6(31.5%)	3(20%)	0.354
Erlotinib	-	2(13.4%)	
**Supportive care**	9(47.5%)	6(40%)	0.545
**NA^b^**	2(10.5%)	3(20%)	-

a NA: not assessable;

b RT: Radiotherapy.

* non-parametric Mann-Whitney test.

## DISCUSSION

This study was conducted to determine the benefits of vinorelbine as a single agent for switch maintenance in advanced NSCLC patients who were treated with first-line gemcitabine plus carboplatin. The results from other single agent maintenance therapy trials have been varied, and thus, provided the idea of this current study. This randomized, prospective, phase 2, single blind clinical trial indicated that vinorelbine maintenance therapy following first-line gemcitabine and carboplatin did not improve PFS or OS in advanced NSCLC but had tolerable toxicity.

Use of cytotoxic agents for switch maintenance therapy in NSCLC has been studied (10–14). Switch maintenance strategies hope to delay the development of resistance to treatment by using a new agent with a different mechanism of action (25). Potentially, switch maintenance therapy may have positive effects on cancers. Tumors would be more sensitive to a different agent at the time of maximum tumor shrinkage than they would be at the time of progression as the Norton-Simon hypothesis states that tumors have populations of faster-growing cells, which are sensitive to therapy, and slower growing, cells that are more resistant (26,27). On the other hand, the use of different and non-cross resistant regimens are required to achieve maximum antitumor effects and suppression of disease progression. As the Goldie-Coldman theory, resistant colons of cancer cells that remain after the initial chemotherapy, can produce disease progression; therefore, best chance of cure or disease relapse prevention would be to use all effective chemotherapy drugs (28).

In the SATURN study (29), a significant increase in OS was observed only in patients who showed SD response after first-line chemotherapy. In our study too, PFS was longer in patients who had SD response to first-line chemotherapy and received switch maintenance chemotherapy with vinorelbine than in patients who had SD response and were in the other group, but the difference was not significant. The findings of the SATURN study suggest that SD might actually be progressive SD and early maintenance therapy actually is a part of early second line chemotherapy.

Westeel et al. (10) showed no PFS benefit of vinorelbine maintenance therapy in NSCLC after platinum-based initial chemotherapy, which is partially similar to our findings. However, in our study anemia was higher but incidences of grade 3, 4 leukopenia, thrombocytopenia, sepsis, pulmonary toxicity, and neuropathy were less. Probably administration of vinorelbine on day 1 and 15 instead of weekly (as in the Westeel et al. study) decreased toxicity. In our study, the toxicity of vinorelbine caused the treatment to be stopped only in 5.2 % of the patients in the maintenance group, whereas in other studies it was stopped in 3.2%, 8%, and 21% of the patients. (30, 31, 10)

Currently, in two studies evaluated maintenance therapy with vinorelbine. In the first study (32) switch maintenance therapy with oral vinorelbine and bevacizumab was given after first-line chemotherapy with cisplatin, gemcitabine, and bevacizumab in patients with advanced stage NSCLC. Improvement in PFS and OS was evident in this study. Another study had similar results for maintenance with oral vinorelbine after first-line treatment with oral vinorelbine plus cisplatin for advanced NSCLC (33). Rubio et al. compared pemetrexed vs. vinorelbine as maintenance therapy after pemetrexed based and vinorelbine based initial treatments. No significant difference was observed between the two groups (34).

Pemetrexed, docetaxel, and gemcitabine all prolong PFS when administrated as maintenance therapies after first-line chemotherapy for NSCLC (35). Numerous meta-analyses have been performed in recent years to study the survival benefits from maintenance strategies (36, 37). Significant increases in OS and PFS have been reported especially with switch maintenance therapies. However, it is necessary to consider that in most developing countries newer cytotoxic agents such as pemetrexed or molecular targeted agents such as erlotinib are expensive or not fully funded by public health care system as maintenance therapy. Besides, we propose that the effectiveness of other targeted maintenance therapies such as cetuximab (38, 39), erlotinib (40), bevacizumab (41), and gefitinib (42) be evaluated in the real world settings. Factors such as ministerial (availability and approval of brand and/or generic cytotoxic agents, and their coverage by insurances), institutional (patients load and turnover, personnel shortages, the cost of administration and hospitalization) should be taken into account. In addition, patient factors (comorbid diseases, performance status, out of pocket cost, patient’s discretion on anticipated adverse events secondary to chemotherapy protocol, convenience of protocol schedules) and physician’s discretion (familiarity with protocol, management of its adverse effects, and consideration of aforementioned factors all together) should also be considered. This study is important for developing countries, where potential toxicities of therapies and their managements are major concerns for clinicians who treat patients with advanced NSCLC with palliative intent. In these countries, specialized centers, as well as expert medical staff and physicians, are not available to deal with most toxicities from chemotherapy. For this reason, we chose vinorelbine for this study because it was available and has toxicities that are more manageable.

In this study, the primary end point was PFS because few studies report significant differences in OS with maintenance therapy. Additionally, the major goal of maintenance therapy is to delay disease progression; the clinically meaningful benefit can be assessed by PFS, which is not influenced by other lines of therapy. Several studies including ours show that PFS was prolonged, but it was not statistically significant. Sample size plays an important role in achieving clinically significant results (43).

## CONCLUSION

Switch maintenance therapy with vinorelbine did not improve PFS in patients with advanced stage NSCLC but had tolerable toxicity. The results of this study might be different from the results reported from highly selected patient populations in phase II or III clinical trials, conducted in developed countries. The importance of these issues will be more evident when we consider the diversity of treatment facilities, trained staff, financial constraints, or even patients’ culture as confounding factors that could have an impact on the selection of systemic treatment in patients with NSCLC.
